# Postoperative Curvature Loss in Three‐Level Anterior Cervical Discectomy and Fusion With Zero‐Profile Device

**DOI:** 10.1111/os.70368

**Published:** 2026-07-26

**Authors:** Zhihao Liu, Xiaqing Sheng, Chengyi Huang, Tingkui Wu, Kangkang Huang, Beiyu Wang, Chen Ding, Ying Hong, Yang Meng, Hao Liu

**Affiliations:** ^1^ Department of Orthopaedic Surgery West China Hospital, Sichuan University Chengdu Sichuan China; ^2^ Trauma Medical Center, Department of Orthopedics Surgery West China Hospital, Sichuan University Chengdu China; ^3^ Department of Orthopedics Orthopedics Research Institute, West China Hospital, Sichuan University Chengdu China; ^4^ West China School of Nursing, Sichuan University Chengdu Sichuan China; ^5^ Department of Anesthesia and Operation Center West China Hospital, Sichuan University Chengdu Sichuan China

**Keywords:** 3‐level cervical disc degenerative disease, anterior cervical discectomy and fusion, curvature, neck pain, T1 slope, zero‐profile

## Abstract

**Purpose:**

Cervical curvature loss is a frequent complication following 3‐level anterior cervical discectomy and fusion (ACDF) using a Zero‐Profile device. Consequently, the capacity of this device to maintain cervical sagittal alignment in 3‐level ACDF remains highly controversial. This study aimed to identify potential predictors for postoperative curvature loss (PCL) and evaluate its impact on clinical outcomes.

**Methods:**

A total of 113 patients who underwent ACDF for 3‐level cervical degenerative disc disease (CDDD) between January 2021 and December 2023 were retrospectively reviewed. Demographic data, radiological parameters, and clinical outcomes were analyzed. Radiographic measures included cervical curvature, T1 slope, C2–7 sagittal vertical axis, and titanium plate and endplate (TPE) distance. Clinical outcomes were assessed using the Visual Analog Scale (VAS), Neck Disability Index (NDI), and Japanese Orthopaedic Association (JOA) scores. Statistical analyses were performed using paired and independent *t*‐tests, as well as Pearson correlation coefficients.

**Results:**

The average curvature loss was 6.82° from 1 week postoperatively to the final follow‐up (*p* < 0.001). However, the final curvature (11.65°) was maintained, representing a 4.33° improvement compared to preoperative values. Significant correlations were observed between PCL and preoperative curvature (*r* = −0.368, *p* = 0.013), preoperative T1 slope (*r* = −0.546, *p* < 0.001), ∆T1 slope (*r* = 0.443, *p* = 0.002), and ∆TPE distance (*r* = 0.417, *p* = 0.004). PCL did not correlate with Japanese Orthopaedic Association (JOA) scores or arm VAS scores at the final follow‐up. Nevertheless, patients with a PCL ≥ 6° exhibited significantly higher neck VAS (*p* = 0.028) and NDI scores (*p* = 0.041).

**Conclusion:**

Although contiguous 3‐level ACDF with a Zero‐Profile device may result in PCL, it preserves an improved cervical lordosis compared to the preoperative baseline. Low preoperative curvature and a low preoperative T1 slope are potentially predictive factors for PCL. Postoperative changes in TPE distance and T1 slope are significantly associated with PCL, suggesting a potential biomechanical link that requires direct validation. Furthermore, PCL may lead to higher neck VAS and NDI scores. Consequently, the Zero‐Profile device may require careful consideration in 3‐level CDDD patients presenting with low preoperative curvature and a low T1 slope. Importantly, the 6° PCL threshold identified is preliminary and requires prospective validation before clinical application.

## Graphical abstract

This study focused on postoperative curvature loss (PCL) for three‐level anterior cervical discectomy and fusion (ACDF) with Zero‐Profile device. Lower preoperative curvature and preoperative T1 slope were predictive factors of PCL. Postoperative change of TPE distance and T1 slope were risk factors. PCL may impair patients' quality of life.

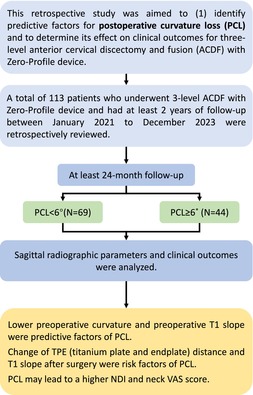

**Abbreviations**
ACDF: Anterior cervical discectomy and fusion
CDDD: Cervical Degenerative Disc Disease
CL: cervical lordosis
cSVA: C2‐7 sagittal vertical axis
FSU: Functional spinal unit
JOA: Japanese Orthopaedic Association
NDI: Neck disability index
PCL: Postoperative curvature loss
T1S: T1 slope
TPE: Titanium plate and endplate
VAS: Visual analogue scale score

## 1 Introduction

Anterior cervical discectomy and fusion (ACDF), first described by Smith and Robinson [1] and popularized by Cloward in the 1950s, has been a standard procedure for treating cervical degenerative disc disease (CDDD). The traditional surgical method uses an anterior plate with a cage for fixation and fusion, which has demonstrated excellent clinical outcomes. However, previous studies have shown that anterior plates are associated with risks such as dysphagia, increased operative time, and adjacent segment ossification [2, 3, 4, 5]. In recent years, the Zero‐Profile (Zero‐P) stand‐alone device has been developed to mitigate these complications. Previous studies [6, 7] have shown that both single‐level and 2‐level Zero‐P yield comparable clinical results and neurological recovery to plate fixation, while also offering shorter operation times, less blood loss, and a lower incidence of adjacent segment degeneration [8]. Several studies [9, 10, 11] have demonstrated that Zero‐P devices are associated with reduced soft tissue edema, a lower incidence of dysphagia, and similar clinical outcomes. However, due to the absence of an anterior plate, several studies speculate that this device may predispose patients to postoperative curvature loss (PCL) [7].

Cervical lordosis plays a crucial role in maintaining normal spinal alignment and stability [12, 13]. Loss of cervical curvature is more likely to accelerate disc degeneration [14]. Numerous studies have reported that curvature loss may lead to neck pain, cervical spinal dysfunction, and altered spinal biomechanics [15, 16, 17]. With an aging population, the demand for 3‐level ACDF is increasing. The capacity of the Zero‐P device to maintain physiological cervical curvature, particularly following 3‐level ACDF, remains controversial. A systematic review [18] found Zero‐P was able to maintain lordosis in 3‐level ACDF while Sheng et al. [19] reported contrasting findings.

Therefore, we aimed to investigate whether cervical curvature can be maintained, identify factors associated with PCL, and evaluate its impact on clinical outcomes in patients undergoing three‐level ACDF with Zero‐P.

## 2 Materials and Methods

This study was approved by the Medical Ethics Committee of our hospital (Ethics Approval Number: 2019946). All patients provided informed consent for the analysis of their clinical data.

### 2.1 Patient Selection

This single‐center, retrospective study included patients treated for CDDD with 3‐level ACDF using the Zero‐P device. A total of 113 consecutive patients underwent surgery from January 2021 to December 2023. Inclusion criteria consisted of: (1) patients with 3‐level radiculopathy or spondylotic myelopathy (e.g., upper or lower limb pain or numbness, neck pain, weakness, and/or dizziness); (2) spinal cord or nerve root compression visible on recent MRI; (3) unsatisfactory response to conservative treatment for at least 3 months; (4) a minimum follow‐up of 24 months; (5) 3‐level ACDF with Zero‐P device implanted at C3/4, C4/5, C5/6 or C4/5, C5/6, C6/7 intervertebral space. Exclusion criteria consisted of: (1) developmental stenosis; (2) history of cervical spine surgery; (3) other cervical diseases including infection, tumor, fracture or other severe systemic diseases.

### 2.2 Surgical Technique

All operations were performed by the same senior spine surgeon. After general anesthesia, the patient was positioned with the neck in a neutral position. A standard right‐sided anterior cervical approach was used. First, complete discectomy was performed at all target levels with removal of disc tissue, posterior longitudinal ligament, and osteophytes to achieve neural decompression. After complete hemostasis, appropriately sized Zero‐P devices (Synthes GmbH, Switzerland) were implanted at the target levels. Next, four locking screws were tightened to fix every Zero‐P device. C‐arm fluoroscopy was used to confirm appropriate device size, position, and reliable fixation. Finally, a drain was inserted before wound closure. The drain was removed 2 days postoperatively. All patients wore a neck brace for 3 months.

### 2.3 Radiological Parameters

**FIGURE 1:**
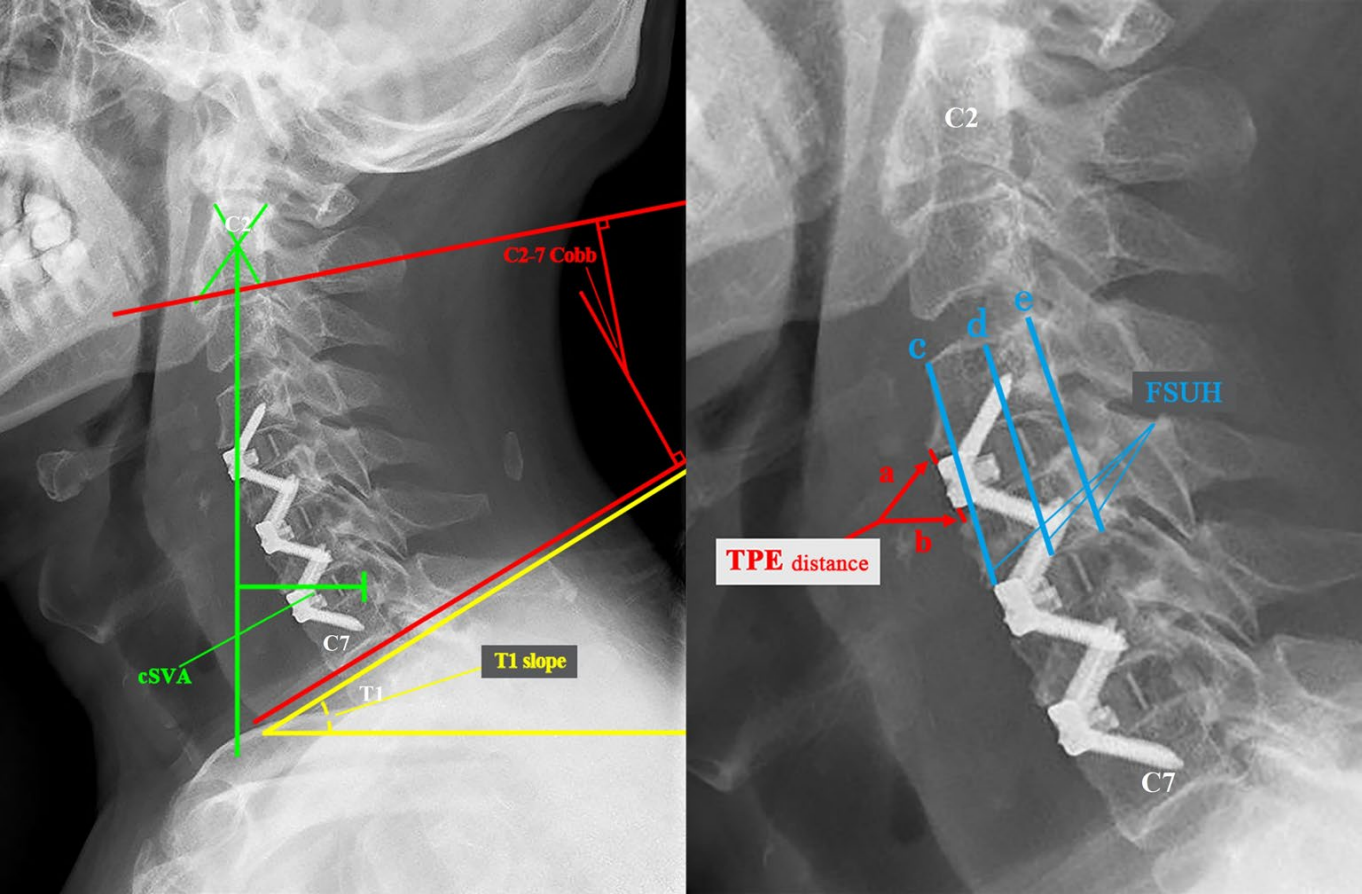
Radiologic parameters. C2‐7 Cobb (angle between lines parallel to the lower endplates of C2 and C7), cSVA (distance between C2 plumb line and superior posterior endplate of C7), T1 slope (angle between superior endplate of T1 and horizontal line), TPE distance (sum of distances between anterior edge of titanium plate and anterior edge of endplate at upper, middle, and lower fusion segments; TPE distance = a + b), FSUH (average height of anterior, middle, and posterior part of fusion segment unit, FSUH = (c + d + e)/3).

Radiographs were obtained preoperatively, at 1 week, 3 months, 6 months, 1 year postoperatively, and at the final follow‐up. Radiological parameters included the C2‐7 Cobb angle (angle formed by the lines parallel to the lower endplates of C2 and C7), C2‐7 sagittal vertical axis (cSVA) (distance between the C2 plumb line and the superior posterior endplate of C7), T1 slope (angle between the superior endplate of T1 and the horizontal line), fusion segment unit height (FSUH, average height of the anterior, middle, and posterior parts of the fused segment), Titanium plate and endplate (TPE) distance (sum of distances between the anterior edge of the titanium plate and the anterior edge of the endplate at the upper, middle and lower fusion segments) (Figure 1). Curvature was measured by C2‐7 Cobb angle. PCL was defined as the absolute value of the difference between the 1‐week postoperative curvature and curvature at subsequent follow‐ups. ∆cSVA was calculated as 1‐week postoperative cSVA minus final follow‐up cSVA. ∆T1 slope was calculated as 1‐week postoperative T1 slope minus final follow‐up T1 slope. ∆TPE distance was calculated as 1‐week postoperative TPE distance minus final follow‐up TPE distance. Subsidence was defined as a loss of FSUH greater than 2 mm from 1 week postoperatively to the final follow‐up. Radiological evaluation was performed by two independent spine surgeons, and the mean values were used for statistical analysis.

### 2.4 Clinical Outcomes

Clinical outcomes were evaluated preoperatively, at 1 week, 3 months, 6 months, 12 months postoperatively, and at the final follow‐up using the JOA, NDI, neck and arm VAS scores to assess recovery from neck pain and radiculopathic symptoms, as well as the ability to return to normal activities. Clinical evaluation was performed in a blinded manner by three spine surgeons not involved in the surgical procedures.

### 2.5 Statistical Analysis

The Kolmogorov–Smirnov test was used to assess whether PCL followed a normal distribution. Pearson correlation coefficients were used to evaluate relationships between PCL at the last follow‐up and potential associated factors. Chi‐square test was used for categorical variables. Differences between groups at the same time point were analyzed by the unpaired Student's *t*‐test. Comparisons of continuous variables between preoperative and postoperative time points were conducted using paired‐sample *t*‐tests. To evaluate the inter‐observer reliability, the intraclass correlation coefficient (ICC) was calculated based on a two‐way random‐effects model with absolute agreement. All analyses were performed using IBM SPSS Statistics, version 26.0 (IBM Corp., Armonk, New York, USA). All *p* values were two‐sided, with statistical significance set at *p* < 0.05.

## 3 Results

### 3.1 Demographics and Baseline Characteristics

A total of 113 patients (Table 1) with CDDD who underwent ACDF were included, comprising 52 females and 61 males, with a mean age of 61.93 ± 10.52 years. The average follow‐up time was 30.38 ± 3.87 months. Patient demographics and PCL occurrence are summarized in Table 1.

**TABLE 1:** Patient demographics and postoperative curvature loss.

Variable	Value
Number of patient	113
Gender	
Male	52 (46.0%)
Female	61 (54.0%)
Age (years)	61.93 ± 10.52
Follow‐up (month)	30.38 ± 3.87
Operation segment	
C3/4, C4/5, C5/6	38 (33.6%)
C4/5, C5/6, C6/7	75 (66.4%)
Operation time (min)	154.62 ± 47.85
Bleeding (mL)	98.08 ± 26.54
PCL appearing	113 (100%)
Final PCL (°)	6.82 ± 3.46
TPE distance appearing	92 (81.4%)
Subsidence	18 (15.9%)

Abbreviations: PCL, postoperative curvature loss; TPE distance, distance between titanium plate and endplate.

### 3.2 Radiological Outcomes and PCL

**FIGURE 2:**
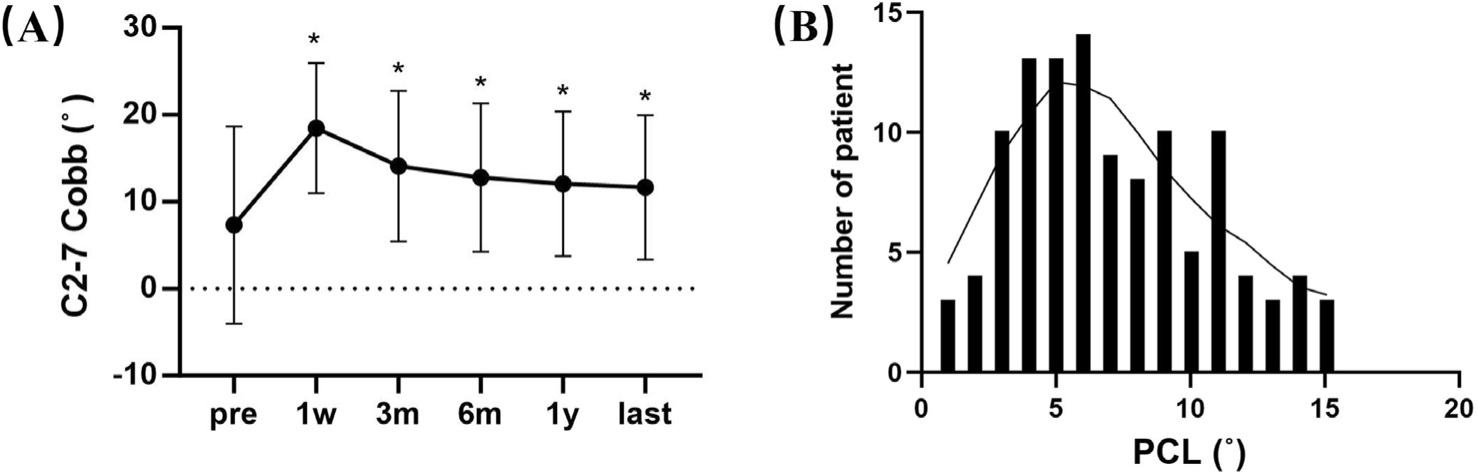
Graph showing the overall change in cervical spine after 3‐level ACDF with Zero‐P device. (A) C2‐7 Cobb peaked at 1‐week postoperation, gradually decreasing to 11.7°. * indicates significant difference compared to 1 week postoperation. (B) PCL distribution (x‐axis, in degree) of the cohort (y‐axis, number of patients). PCL range from 1.24° to 15.19°, and most patients lost 5°–7° at the last follow‐up.

**FIGURE 3:**
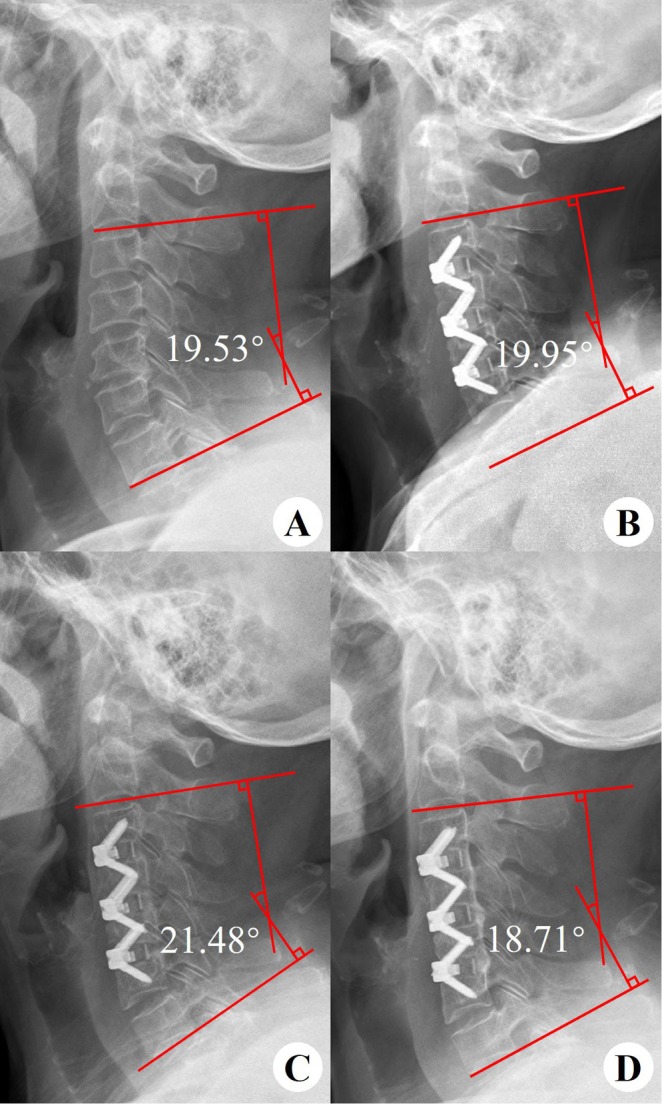
A 71‐year‐old woman with 3‐level CDDD, 2 years after ACDF with Zero‐p device. (A) Preoperative radiograph shows C2‐7 Cobb angle of 19.53°. (B) Radiograph at 1‐week post‐operation shows C2‐7 Cobb angle of 19.95°. (C) Radiograph at 3 months post‐operation shows C2‐7 Cobb angle of 21.48°. (D) Radiograph at 25 months post‐operation shows curvature loss of 1.24° compared to early post‐operation, but maintained at 18.71°.

The inter‐observer agreement for all radiological parameters was excellent, with ICC values ranging from 0.84 to 0.93 (all *p* < 0.01). Cervical curvature demonstrated significant correction at 1 week postoperatively (7.32° ± 11.21° vs. 18.47° ± 7.41°, *p* < 0.05) (Figure 2A). PCL at the final follow‐up ranged from 1.24° to 15.19° (Figure 2B). Despite this loss, the final curvature (11.65° ± 8.20°) remained significantly improved (by approximately 60%) compared to preoperative curvature (7.32° ± 11.21°, *p* < 0.05) (Figure 3). To identify potential predictors, patients were divided into two groups based on PCL at final follow‐up (PCL < 6° and PCL ≥ 6°). No significant difference was found in age, gender, follow‐up time, or operation segment distribution between two groups (Table 2). Significant differences were observed in preoperative curvature, preoperative T1 slope, and ∆T1 slope between groups. Pearson regression analysis revealed significant correlations between PCL and preoperative curvature (*r* = −0.368, *p* = 0.013), preoperative T1 slope (*r* = −0.546, *p* < 0.001), ∆T1 slope (*r* = 0.443, *p* = 0.002), ∆TPE distance (*r* = 0.417, *p* = 0.004) (Table 3). A measurable TPE distance was observed in 92 patients (81.4%) postoperatively, and this gap was found to be decreased at the final follow‐up.

**TABLE 2:** Comparison of patient demographics and sagittal alignment between two groups.

Variable	PCL < 6°	PCL ≥ 6°	*p*
Number of patient	69	44	
Age (years)	63.92 ± 9.97	59.67 ± 10.66	0.184
Gender			0.066
Male	27 (39.1%)	25 (56.8%)	
Female	42 (60.9%)	19 (43.2%)	
Follow‐up (months)	30.13 ± 3.26	30.71 ± 4.39	0.617
Operation segment			0.368
C3/4, C4/5, C5/6	21 (30.4%)	17 (38.6%)	
C4/5, C5/6, C6/7	48 (69.6%)	27 (61.4%)	
Pre CL (°)	11.34 ± 8.73	−2.73 ± 11.94	0.009**
Preoperative cSVA (mm)	18.81 ± 10.32	16.23 ± 8.57	0.382
∆cSVA (mm)	2.36 ± 8.59	−1.44 ± 7.12	0.125
Preoperative T1S (°)	26.21 ± 6.31	18.96 ± 5.74	< 0.001**
∆T1S (°)	5.32 ± 3.02	7.53 ± 4.13	0.049*
TPE distance (mm)	2.65 ± 2.30	3.77 ± 2.29	0.635
∆TPE distance (mm)	1.68 ± 1.70	2.80 ± 1.72	0.038*
Subsidence appearing	8	10	0.115

Abbreviations: cSVA, C2‐7 sagittal vertical axis; PCL, postoperative curvature loss; Pre CL, preoperative cervical lordosis; T1S, T1 slope; TPE distance, distance between titanium plate and endplate.

* *p* < 0.05.

** *p* < 0.01.

**TABLE 3:** Correlation analysis between sagittal alignment and PCL.

	PCL	Pre CL	T1S	△T1S	cSVA	△cSVA	TPE distance	△TPE distance
PCL	—							
Pre CL	−0.368*	—						
T1S	−0.546**	0.564**	—					
△T1S	0.443**	−0.218	−0.148	—				
cSVA	−0.250	0.106	0.126	0.059	—			
△cSVA	−0.204	−0.069	−0.019	0.190	0.588**	—		
TPE distance	0.229	−0.085	−0.148	0.161	−0.269	−0.028	—	
△TPE distance	0.417**	−0.223	−0.356*	0.068	−0.380*	−0.137	0.765**	—

Abbreviations: cSVA, C2‐7 sagittal vertical axis; PCL, postoperative curvature loss; Pre CL, preoperative cervical lordosis; T1S, T1 slope; TPE distance, distance between titanium plate and endplate.

* *p* < 0.05.

** *p* < 0.01.

### 3.3 Clinical Outcomes and Complications

**FIGURE 4:**
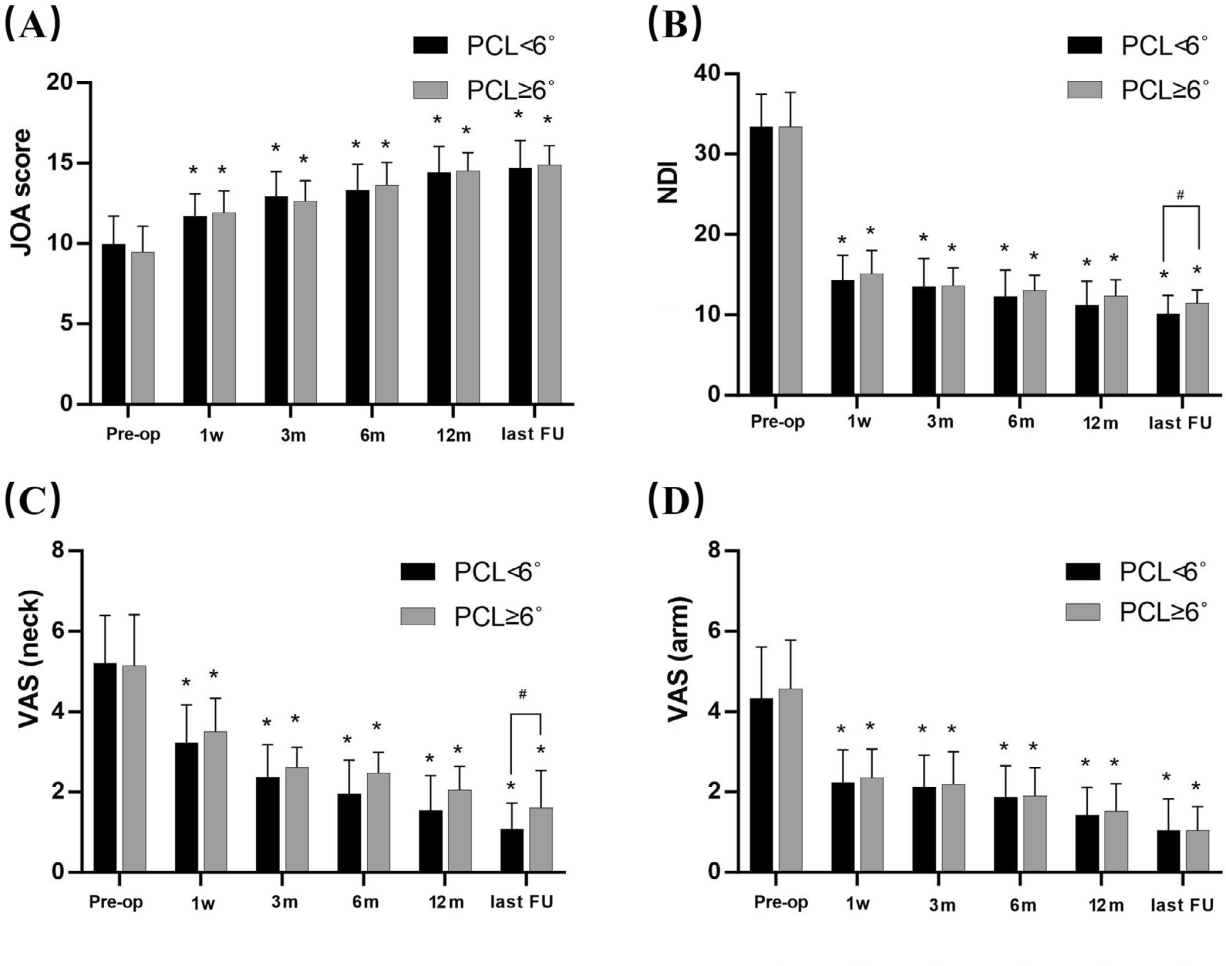
Graph showing clinical outcomes from preoperation to the last follow‐up. *Significant improments were observed at any follow‐up time in (A) JOA score, (B) NDI, (C) VAS (neck) and (D) VAS (arm). ^#^A significant difference was found between two groups.

Regarding clinical outcomes, all patients were discharged as planned, and none required revision surgery. Although prosthesis subsidence occurred in 18 patients, none exhibited neurological symptoms or required reoperation. Clinical outcomes showed significant improvement postoperatively in both groups as shown in Figure 4. Patients with PCL < 6° had lower neck VAS scores (1.08 ± 0.64 vs. 1.62 ± 0.92, *p* = 0.028) and lower NDI scores (10.08 ± 2.34 vs. 11.43 ± 1.66, *p* = 0.041) at the last follow‐up than those with PCL ≥ 6°. However, arm VAS and JOA scores were comparable between groups at any follow‐up time.

## 4 Discussion

In this study, we found that contiguous 3‐level ACDF with the Zero‐P device significantly improved cervical lordosis compared to the preoperative state, despite the common occurrence of PCL. Furthermore, we identified low preoperative curvature and a low T1 slope as predictive factors for PCL, while postoperative changes in the T1 slope and TPE distance were associated with PCL. Clinically, PCL was found to correlate with more severe postoperative neck pain (VAS) and disability (NDI).

### 4.1 Curvature Correction and Maintenance

Loss of cervical lordosis may cause debilitating neck pain and disability, as well as alter overall spinal biomechanics [20]. 3‐level ACDF with Zero‐P can correct and partly restore overall curvature. Curvature decreased by approximately 6.82° from 1 week postoperatively to the last follow‐up but improved by 4.33° compared with preoperative values, consistent with previous findings. Sheng et al. [19] reported curvature of approximately 20° at 3 months postoperation, declining to about 16° at final follow‐up, representing an overall decrease of about 4.5°. He et al. [21] followed patients undergoing consecutive three‐level ACDF with either Zero‐profile or plate. Cervical curvature was maintained at 18.06° and 19.17°, respectively, at 1‐year follow‐up, declining to 16.35° and 17.98° at 5‐year follow‐up, both demonstrating significant improvement compared to preoperative values. Chen et al. [22] reported a 26.8% curvature loss at the last follow‐up compared to early postoperation, with the plate group maintaining 4° greater curvature than the Zero‐P group. Based on our findings and the existing literature, 3‐level ACDF using a Zero‐P device effectively corrects initial cervical curvature, even if slight loss occurs over time.

### 4.2 Predictors of PCL

PCL is associated with preoperative curvature and preoperative T1 slope. To date, no study has explored the relationship between PCL and preoperative curvature or preoperative T1 slope following 3‐level Zero‐P ACDF. Our correlation analysis found that lower preoperative curvature and lower preoperative T1 slope were significantly correlated with greater PCL. We postulate that PCL represents a biomechanical adaptation to the patient's original spinal alignment and natural degenerative cascade. This may be influenced by the patient's cervical spine physiology, surrounding muscles, ligaments, soft tissues, and daily habits. Although cervical curvature is corrected after surgery, surrounding soft tissues, muscles, and ligaments remain largely unchanged. Consequently, mechanical forces may still alter cervical curvature before fusion. Therefore, we hypothesize that lower preoperative curvature is related to greater PCL. Regarding the T1 slope, Kwon et al. [23] reported a similar correlation between PCL and T1 slope in a 6‐month follow‐up study. T1 is a transitional vertebra at the cervicothoracic junction, closely associated with cervical lordosis and horizontal gaze stability. A higher T1 slope requires compensatory cervical flexion to maintain horizontal alignment of the upper cervical spine [24]. Conversely, a lower T1 slope may be associated with reduced cervical curvature. As shown in Table 3, the T1 slope was positively correlated with preoperative cervical curvature, which may explain its correlation with PCL. Patwardhan et al. [25] conducted a biomechanical study concluding that increased C2–C7 SVA leads to flexion of lower cervical segments and hyperextension of suboccipital segments to maintain horizontal gaze. However, in our study, neither preoperative cSVA nor ∆cSVA was related to PCL.

### 4.3 Impact of TPE Distance

After Zero‐P prosthesis implantation, we observed a gap between the endplate and the titanium plate at the anterior vertebral body in most patients. To our knowledge, this is the first study to specifically analyze the association between TPE distance and PCL. The arc‐shaped endplate may prevent perfect cage fit within the intervertebral space. To increase the fusion contact area and improve prosthesis placement, the anterior part of the endplate is typically ground, enhancing contact between the posterior part of the endplate and prosthesis. However, this surgical modification unavoidably increases the space between the anterior edge of the prosthesis and the endplate, defining the TPE distance. A finite element study showed that Zero‐P devices increased strain and stress on bone grafts, with higher stress and larger predicted effective strain area at the graft‐endplate interface, promoting bone growth in fused segments [26]. Deng et al. [27] found that TPE distance accelerated fusion but sacrificed cervical lordosis and segmental cervical angle. Under physiological compressive loads, this anterior gap gradually collapses, resulting in localized anterior column height loss, which may contribute to PCL. We examined 339 intervertebral spaces in 113 patients. TPE distance was observed in 92 patients (81.4%) after surgery, and almost all distances decreased at the last follow‐up, supporting our hypothesis.

### 4.4 PCL and Clinical Outcomes

There was a significant difference between the two groups in neck VAS and NDI scores. Neck pain may result from nerve compression, peripheral soft tissue strain, or chronic inflammation. Low curvature leads to local stress changes in the cervical spine, potentially causing soft tissue congestion and strain, leading to neck pain. Liu et al. [28] suggested that low cervical curvature significantly impacts the cervical mechanical environment and contributes to neck pain. McAviney et al. [29] found patients with kyphosis were 18 times more likely to experience cervical symptoms, identifying it as significantly associated with neck pain. Zhu et al. [30] found low curvature was related to neck VAS but not JOA scores, consistent with our results. Similar findings are reported in other studies [25, 31, 32, 33]. The NDI scale aims to evaluate the impact of neck disability on daily life and work. Xu et al. [34] found that reduced cervical lordosis was significantly associated with poor NDI scores, aligning with our results.

### 4.5 Strengths and Limitations

This study benefits from a single‐center design with all procedures performed by a single surgeon, which ensured high technical consistency and minimized surgical confounding, but limited the external validity and generalizability of our results to other institutions. Crucially, it is the first to investigate the relationship between TPE distance and PCL. However, there are several limitations. First, the retrospective design carries inherent risks of selection bias and unmeasured confounding variables, such as patient compliance with postoperative rehabilitation exercises. Additionally, we lacked data on potential confounders including bone mineral density (BMD), smoking status, and body mass index (BMI), which could influence both TPE distance collapse and PCL. Second, the relatively short follow‐up period may be insufficient to fully evaluate long‐term complications such as adjacent segment disease (ASD) or implant subsidence; extended follow‐up studies are necessary. Third, we utilized a cut‐off of 6° to categorize PCL. As there is currently no universally established minimal clinically important difference (MCID) for cervical lordosis measured in degrees, this threshold serves only as preliminary evidence. Future longitudinal studies are needed to determine the magnitude of change in cervical alignment that constitutes a clinically meaningful difference for patients. Fourth, while our radiographic data strongly support the mechanical compression theory of the TPE distance, direct biomechanical evidence is currently lacking. We plan to conduct dedicated finite element analysis studies to validate this theoretical mechanism. Fifth, we assumed the 1‐week postoperative alignment represents the true functional correction, which may still be subject to early positional variation. Finally, additional cervical sagittal parameters, such as the Chin‐Brow Vertical Angle (CBVA) [35], were not assessed, which may limit the comprehensiveness of our findings. Future prospective, multicenter studies incorporating a broader set of radiographic parameters are warranted to validate and expand upon our findings.

## 5 Conclusions

Contiguous 3‐level ACDF with a Zero‐Profile device achieves significant improvement in cervical lordosis compared to the preoperative state, despite the common occurrence of PCL. This study identifies low preoperative curvature and a low T1 slope as key predictive factors for PCL. And postoperative changes in the T1 slope and TPE distance may associate with PCL, requiring direct biomechanical validation. Clinically, PCL correlates with more severe postoperative neck pain (VAS) and disability (NDI). Therefore, surgeons should exercise caution and consider sagittal parameters when selecting the Zero‐P device for 3‐level fusion. The 6° threshold identified in this study is preliminary and should not be used to guide clinical decision‐making until validated in future prospective studies.

## Author Contributions

**Zhihao Liu:** writing – original draft, writing – review and editing, data curation. **Xiaqing Sheng:** writing – review and editing, data curation. **Chengyi Huang:** data curation, writing – review and editing. **Tingkui Wu:** writing – review and editing, data curation. **Kangkang Huang:** data curation. **Ying Hong:** methodology. **Yang Meng:** conceptualization, funding acquisition. **Beiyu Wang:** methodology. **Chen Ding:** conceptualization, funding acquisition. **Hao Liu:** funding acquisition, conceptualization, methodology.

## Funding

This study was supported by the 1·3·5 project for disciplines of excellence–Clinical Research Fund, West China Hospital, Sichuan University (2025HXFH037), Natural Science Foundation of Sichuan Province (2025ZNSFSC1786), Sichuan Province Science and Technology Support Program of China (2024YFHZ0163), Clinical New Technology Fund, West China Hospital, Sichuan University (25HXJS027). No relevant financial activities outside the submitted work.

## Ethics Statement

This study was performed in line with the principles of the Declaration of Helsinki. Approval was granted by the Ethics Committee of West China Hospital, Sichuan University (No. 2019946).

## Consent

Informed consent was obtained from all individual participants included in the study. The authors affirm that human research participants provided informed consent for publication of the images in Figures 1 and 3.

## Conflicts of Interest

The authors declare no conflicts of interest.

## Data Availability

The data that support the findings of this study are available from the corresponding author upon reasonable request.
